# Gestational length assignment based on last menstrual period, first trimester crown-rump length, ovulation, and implantation timing

**DOI:** 10.1007/s00404-016-4153-3

**Published:** 2016-07-28

**Authors:** Amita A. Mahendru, Charlotte S. Wilhelm-Benartzi, Ian B. Wilkinson, Carmel M. McEniery, Sarah Johnson, Christoph Lees

**Affiliations:** 1 Fetal Medicine Department, Nottingham University Hospitals NHS Trust, Nottingham, UK; 2 Department of Fetal Medicine, Addenbrooke’s Hospital, Cambridge, CB2 2QQ UK; 3 ICTU-Cancer Clinical Trials Unit, Department of Surgery and Cancer, Imperial College London, London, UK; 4 Division of Experimental Medicine and Immunotherapeutics, Addenbrooke’s Hospital, University of Cambridge, Box 98, Cambridge, CB20QQ UK; 5 SPD Development Company Ltd., Bedford, UK; 6 Department of Surgery and Cancer, Institute of Developmental and Reproductive Biology, Imperial College London, London, W12 0HS UK; 7 Department of Development and Regeneration, University Hospitals Leuven, KU Leuven, Campus Gasthuisberg, 3000 Leuven, Belgium

**Keywords:** Estimated date of delivery, Ultrasound, Pregnancy dating, CRL

## Abstract

**Purpose:**

Understanding the natural length of human pregnancy is central to clinical care. However, variability in the reference methods to assign gestational age (GA) confound our understanding of pregnancy length. Assignation from ultrasound measurement of fetal crown-rump length (CRL) has superseded that based on last menstrual period (LMP). Our aim was to estimate gestational length based on LMP, ultrasound CRL, and implantation that were known, compared to pregnancy duration assigned by day of ovulation.

**Methods:**

Prospective study in 143 women trying to conceive. In 71 ongoing pregnancies, gestational length was estimated from LMP, CRL at 10–14 weeks, ovulation, and implantation day. For each method of GA assignment, the distribution in observed gestational length was derived and both agreement and correlation between the methods determined.

**Results:**

Median ovulation and implantation days were 16 and 27, respectively. The gestational length based on LMP, CRL, implantation, and ovulation was similar: 279, 278, 276.5 and 276.5 days, respectively. The distributions for observed gestational length were widest where GA was assigned from CRL and LMP and narrowest when assigned from implantation and ovulation day. The strongest correlation for gestational length assessment was between ovulation and implantation (*r* = 0.98) and weakest between CRL and LMP (*r* = 0.88).

**Conclusions:**

The most accurate method of predicting gestational length is ovulation day, and this agrees closely with implantation day. Prediction of gestational length from CRL and known LMP are both inferior to ovulation and implantation day. This information could have important implications on the routine assignment of gestational age.

## Introduction

Gestational age (GA) assignment has, for many centuries, been based on knowledge of a woman’s last menstrual period (LMP). The estimated date of delivery (EDD) commonly known as ‘due date’ has traditionally been calculated from this by adding 280 days to the LMP date, or through Naegele’s rule: subtracting 3 from the month and adding 7 days [1]. This method of dating a pregnancy is inaccurate where there is not a reliable recollection of LMP, or if menstrual cycles are short, long, or irregular. Certainty of LMP has been reported as low as 32 % [2] and the observation of number preference in birth records, for example, 15th of the month being stated 2.5 times more than expected [3] indicates that for many women, LMP is little more than a guess. Even when accurately known, average cycle variability is approximately 7 days [4, 5] mainly due to variation in length of the follicular phase [6], meaning that ovulation leading to pregnancy has been shown to occur anywhere from day 9–30 of the cycle [5]. This introduces a significant error into the use of LMP to assign GA.

Since the description in the early 1970s of ultrasound measurement of fetal crown-rump length (CRL) in the first trimester and the development of charts converting the CRL into assumed length of pregnancy based on LMP [7], assessment of GA by ultrasound has become routine in most countries where pregnancy ultrasound is performed.

In the UK, following guidance from the National Institute of Clinical Excellence (NICE) in 2008 [8], GA is routinely determined by measuring the fetal crown-rump length (CRL) at 10–14 weeks using ultrasound [8, 9]. The CRL charts were constructed from observed first trimester CRL measurements in relation to GA calculated from the last menstrual period (LMP) in women with regular menstrual cycles [7, 10, 11], but do not consider biological variation in fetal size as a result of individual differences in ovulation and implantation timing [7]. Intra-operator variability and inter-operator variability when conducting a scan can also introduce errors, with the SD calculated to be 1.27 and 1.37 days gestation for intra-operator variability and inter-operator variability, respectively [12]. Furthermore, GA assigned by LMP and CRL is based on the assumption that ovulation occurs mid cycle in women with regular menstrual cycles. However, only 10 % of women with a regular 28 day menstrual cycle ovulate on day 14 [13, 14] and the median ovulation day in women with regular cycles is day 16 [15, 16]. Although the date of intercourse may be known in a natural conception, sperm survival times of up to 7 days have been reported in fertile cervical mucus [17], and this translates to conception being possible following intercourse at least 5 days prior to ovulation [18], so intercourse day is not an accurate reference for the start of pregnancy. Day of ovulation provides an excellent reference for the start of pregnancy, as the egg has a survival time of less than a day, but is not generally known in a natural conception. The interval between ovulation and implantation was found to be 8–10 days in 84 % of pregnancies, but could vary by up to 11 days [19], where implantation has been assumed to be represented by human chorionic gonadotrophin being detectable in blood or urine [20].

We have, recently, reported for the first time on the impact of ovulation and implantation timing on the crown-rump length, and hence GA assessment in the first trimester of pregnancy [20]. Using highly sensitive urinary ovulation LH [21] and hCG [19, 22, 23] testing kits, we were able to prospectively detect ovulation and implantation dates with a high degree of accuracy in women planning to conceive in whom LMP was known and first trimester ultrasound CRL measurements made: the timing of ovulation and implantation strongly influenced the size of the fetus at 10–14 weeks independent of LMP. In other words, late ovulation and implantation led to a smaller than expected first trimester CRL and early implantation to a larger than expected CRL at 10-14 weeks. Importantly, very long ovulation to implantation interval is highly related to likelihood of miscarriage [19, 20]. Recently, the length of pregnancy has been found to be more variable when calculated from LMP compared to ovulation timing, though in that study, the authors did not report ultrasound data [24].

Though the effect of ovulation and implantation on fetal size at the first trimester scan may be known, it is not clear what effect ovulation and implantation have on the length of gestation. If implantation and ovulation occur later, this may mean that the pregnancy, as dated from LMP, lasts longer, and vice versa [24]. Fetal size in the first trimester may be a proxy for post-embryonic implantation fetal age, but there again fetal size and gestational length may be independent of each other. The question, therefore, is how closely does predicted gestational length based on LMP and first trimester observed CRL relates to that based on known ovulation and implantation timing? This is important, because all methods of assigning GA, hence predicting length of gestation, have an inherent error and variability. Assigning GA accurately is essential, as this allows early pregnancy ultrasound scans to be interpreted, for Down’s screening based on ultrasound and biochemistry and deviations in fetal growth to be diagnosed. As GA is strongly associated with perinatal outcome [25], it is often, the most important, determinant in making critical clinical decisions regarding pregnancy management at the margins of viability.

We, therefore, compared gestational length when GA was assigned by LMP, ultrasound CRL, ovulation, and implantation day in a cohort of women planning to conceive and assessed both the correlation and the limits of agreement between the different methods.

## Methods

### Study population and participants

We prospectively recruited 143 women trying to conceive via open advertisement in the hospital, GP surgeries, newspapers, pre-school groups, or by invitation letter. This was part of a larger study on cardiovascular changes in pregnancy, and sample size was defined for this larger feasibility study rather than specifically to investigate different methods of gestational age assessment. All women were healthy, non-smokers, and not known to have diabetes, thrombophilia, or fertility problems.

### Ethical approval

The study received ethical approval from the local Research Ethics Committee, and written consent was obtained at the time of recruitment.

## Materials and methods

Age, ethnicity, last menstrual period (LMP), detailed menstrual history, obstetric and cardiovascular history, height, weight, and body mass index (BMI) were recorded at recruitment. The women started using digital urinary home ovulation and pregnancy test kits at least a month after stopping contraception. They were asked to perform daily digital ovulation tests (Clearblue) from the 6th day of their LMP until the urine LH surge was detected and then performed daily pregnancy tests (Clearblue digital tests) from 8 days after the LH surge until they either had three consecutive positive pregnancy tests or their next period. They continued testing in every menstrual cycle until they became pregnant or for up to 6–12 months if no clinical pregnancy occurred. The urinary tests were provided free of charge by SPD Development Company Ltd. (Bedford, UK), and consisted of a re-usable digital reader, with disposable test sticks. This ensured LH and hCG results were not subject to user interpretation, and enabled objective comparison between different volunteers.

A rise in urinary LH predicts ovulation at a mean of 20 h from the initial LH rise [21]. We, therefore, calculated the ‘LH surge + 1 day’ to define the day of ovulation as has been previously described [16, 20, 21]. The day of the first positive pregnancy test using sensitive digital urine pregnancy test kits was reported as the ‘implantation day’ in a similar way to previously described [19]. The tests used have a sensitivity of 50 mIU/ml and are lateral-flow based tests that are directly sampled by the user. The precision of these tests is such that results are consistently “Pregnant” at concentrations of hCG at or above the test sensitivity. As early rise in urinary hCG is extremely consistent and the first positive “Pregnant” result from the home use, digital test can be equated to being 3–4 days from the first appearance of hCG (AutoDELFIA: 0.01 mIU/ml sensitivity, day 8 post ovulation) median hCG concentration and inter-quartile range 0 mIU/ml (0–0.54), day 9; 1.35 mIU/ml (0.28–4.82), day 10; 7.57 mIU/ml (4.1–14.0), day 11; 19.5 mIU/ml (10.4–30.3), day 12; 38.3 mIU/ml (22.7–60.9), day 13; and 69.3 mIU/ml (40.1–106.8) [26].

Ultrasound scans were performed at 10–14 weeks of gestation from either the LMP or ovulation timing. For the purposes of this study, only women with ongoing pregnancies at 10–14 weeks were included. The fetal CRL at 10–14 weeks was measured abdominally in a mid-sagittal plane with the genital tubercle and the fetal spine longitudinally in view and measuring the maximum crown to rump length [27]. The best of three CRL measurements was then taken.

Gestational ages based on LMP, CRL, ovulation, and implantation were then assigned for each pregnancy. From this, estimated gestational length was calculated based on LMP, CRL using Robinson’s charts, and implantation dates. For example, estimated GA based on ovulation was derived by subtracting 14 days from the observed ovulation date (LH +1) to give an ‘effective LMP’ adjusted for ovulation, as is convention for pregnancy dating, for example, in IVF pregnancy. Gestational length based on implantation was derived by subtracting 27 days from the observed implantation date to give an ‘effective LMP’ adjusted for implantation.

### Statistical analyses

The statistical analysis was performed using R version 3.0.1 Core Team (2013), (R: a language and environment for statistical computing. R Foundation for Statistical Computing, Vienna, Austria. URL http://www.R-project.org/). The data were checked for normality of distribution, and data are expressed as mean ± standard deviation and median ± inter-quartile range (IQR) or range as appropriate.

Observed gestational length and absolute mean difference about the median were calculated for gestational length, where GA was assigned from LMP, CRL, ovulation, and implantation.

The Bland–Altman statistic between all methods for observed gestational length based on each method of assigning GA was derived pairwise for all comparisons to evaluate the agreement among two measurement techniques. The Spearman correlation between each method of assigning GA: ovulation day, implantation day, LMP, and CRL was calculated.

## Results

**Fig. 1:**
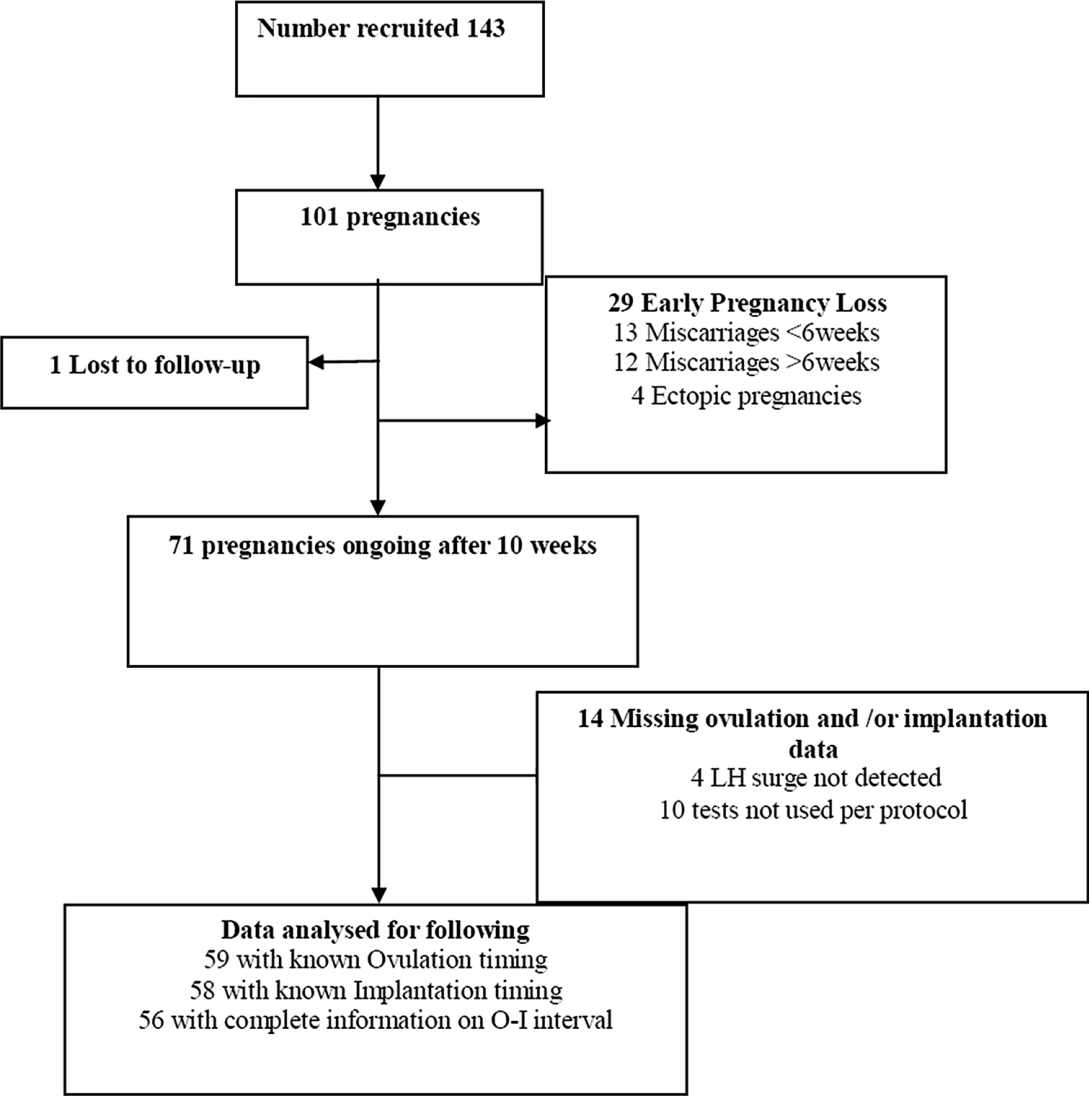
Recruitment flowchart

101 women became pregnant, whilst enrolled in the study (Fig. 1). The median time taken to conceive was 5 (inter-quartile range 2–7) months. Characteristics of the 71 women with ongoing pregnancies at 10–14 weeks are described in Table 1. The median ovulation day, implantation day, and ovulation to implantation interval are described in Table 2. Ovulation leading to pregnancy was as early as day 11 in the cycle and as late as day 39 in this cohort.

**Table 1:** Characteristics of the 71 participants with ongoing pregnancy after 10 weeks

Characteristics	Participants (*n* = 71)
Median (IQR) maternal age, years	32 (29–35)
Ethnicity	
White	65 (91.6)
Black	2 (2.8)
Asian	2 (2.8)
Others	2 (2.8)
Parity	
Nulliparous	37 (52)
Multiparous	34 (48)

Values are numbers (percentages) unless stated otherwise

**Table 2:** Summary of menstrual cycle data of women who became pregnant

Days	*n*	Pregnancies ongoing >10 weeks
Ovulation day	59	16 (11–39)
Menstrual cycle length	69	28 (21–60)
Implantation day	58	27 (23–44)

Values are median (range) unless specified. Ovulation and implantation day relate to the cycle, in which the volunteer achieved pregnancy. Menstrual cycle length is calculated from the study cycles prior to pregnancy. Where women became pregnant in first cycle, no menstrual cycle length data are available

*n* Number where complete information available

**Fig. 2:**
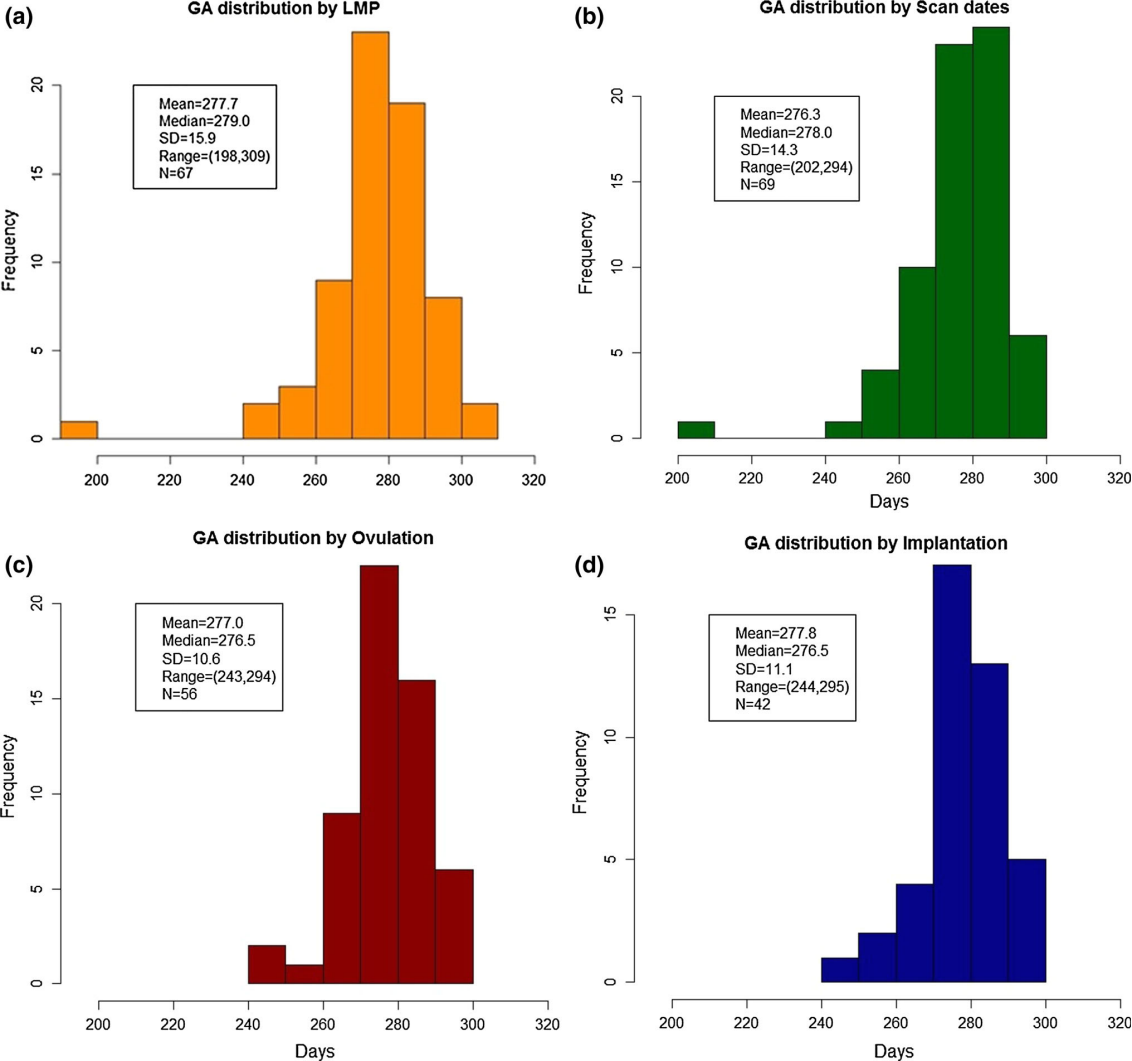
Frequency distributions for gestational age at delivery based on **a** LMP, **b** CRL, **c** ovulation, and **d** implantation

**Fig. 3:**
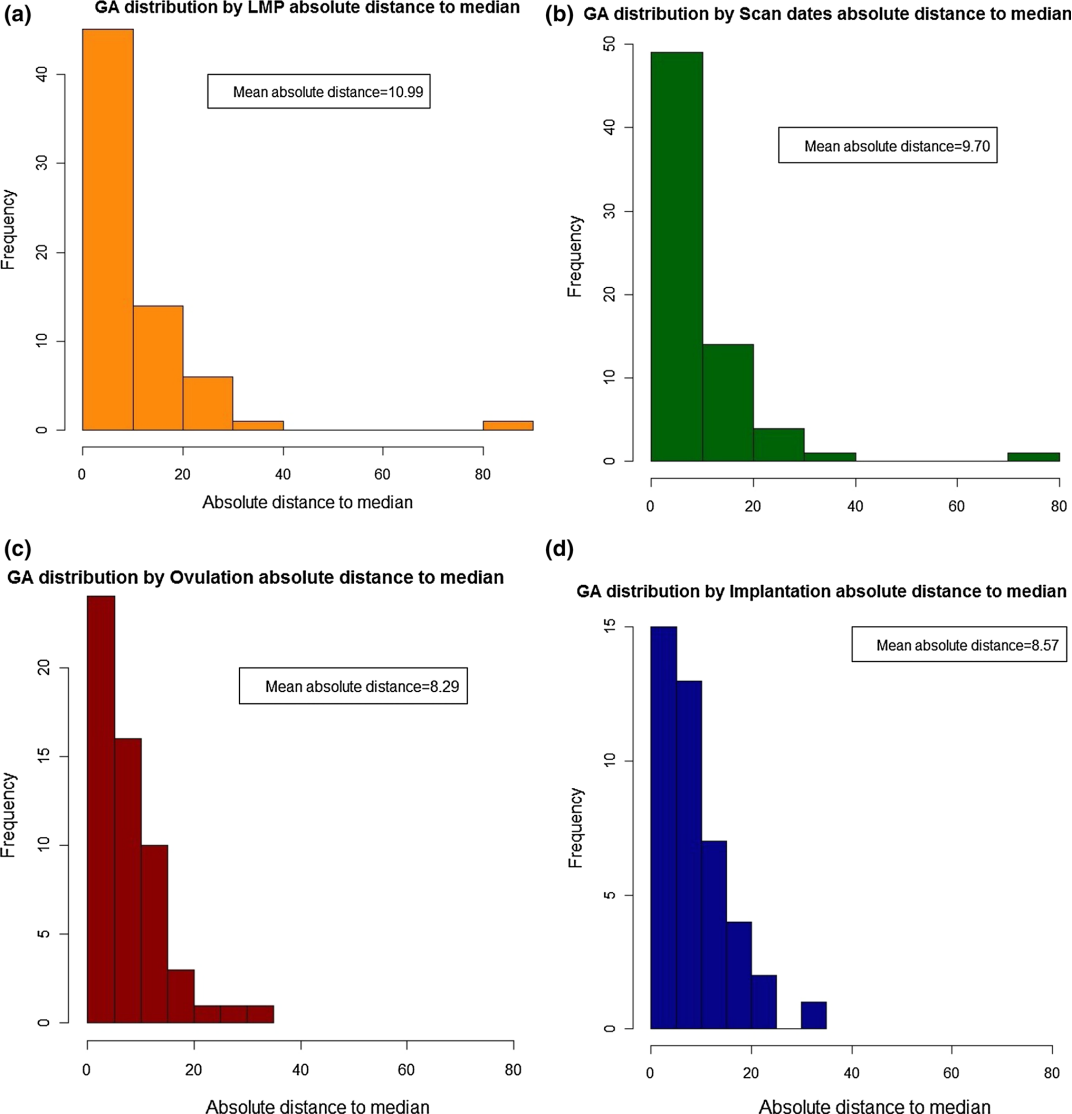
Distributions showing the absolute differences about the median gestational age at delivery based on **a** LMP, **b** CRL, **c** ovulation, and **d** implantation

**Fig. 4:**
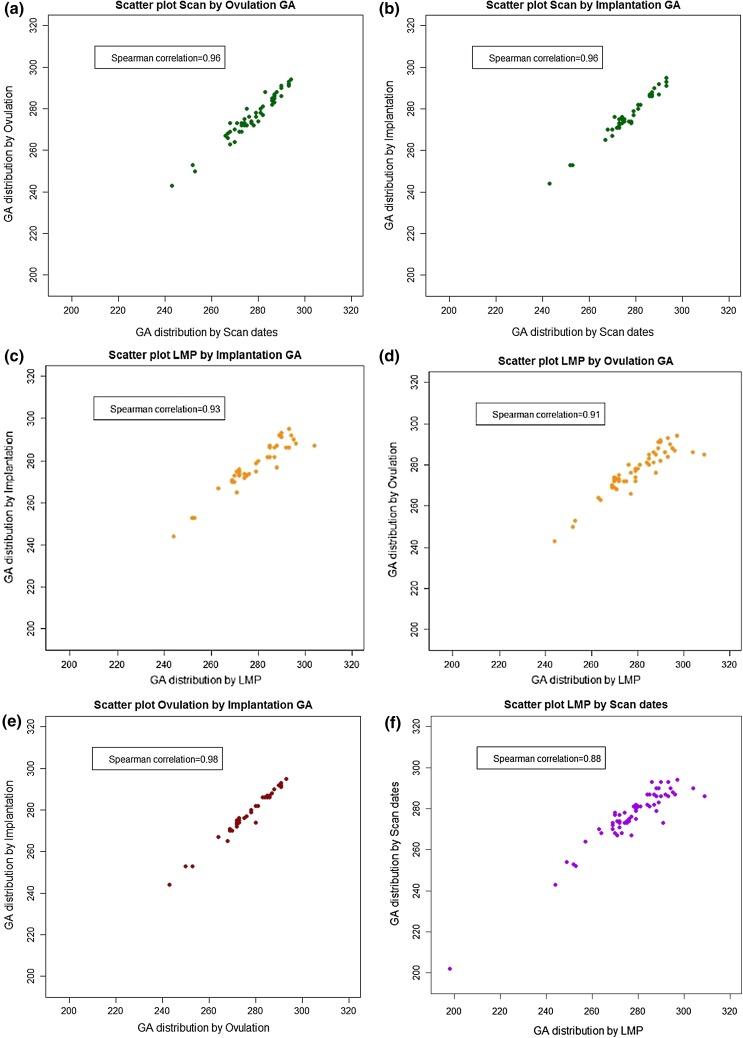
*Scatter plots* and correlation coefficients for **a** ultrasound scan and ovulation, **b** ultrasound scan and implantation, **c** LMP and implantation, **d** LMP and ovulation, and **e** ovulation and implantation and **f** LMP by scan

The frequency distributions for gestational length were widest where GA assignment was based on LMP and ultrasound, and narrowest for implantation and ovulation day where the distributions were similar to one another (Fig. 2). The distributions were similar where the absolute mean differences about the median were considered (Fig. 3). The Bland–Altman analyses showed that the two techniques with the greatest mean difference for determining gestational length were where GA assignation based on LMP and ultrasound CRL were compared; here, the range for limits of agreement (LoA) was 17.3 days. The range for LoA was narrowest (6.24 days), where implantation day and ovulation day were compared (Table 3). The non parametric Spearman correlations between gestational length based on LMP and CRL showed the least strong correlation (0.88) and between implantation and ovulation day the strongest (0.98) (Table 4, Fig 4).

**Table 3:** Bland–Altman pairwise comparisons for gestational length based on the four methods of assigning gestational age in ascending order from smallest limit of agreement

GA comparison	Mean/bias	95 % limit of agreement
Ovulation–implantation	1.17	(−1.95, 4.29)
CRL–implantation	−0.24	(−3.98, 3.51)
Ovulation–CRL	−1.40	(−5.76, 2.95)
Ovulation–LMP	−2.12	(−10.54, 6.30)
LMP–implantation	−0.95	(−9.50, 7.60)
LMP–CRL	0.71	(−7.91, 9.34)

The non-parametric Spearman correlations between gestational length based on LMP and CRL showed the least strong correlation (0.88) and between implantation and ovulation day, the strongest (0.98) (Table 4). All correlations were highly significant at the *p* < 0.05 level.

**Table 4:** Spearman correlations of pairwise comparisons between all combinations of gestational length based on GA assignation from LMP, ultrasound CRL, ovulation day, and implantation day in descending order from highest R

Spearman correlation of GA comparisons	*p* values	*R*
Ovulation–implantation	<2.2e−16	0.98
CRL–implantation	<2.2e−16	0.96
Ovulation–CRL	<2.2e−16	0.96
LMP–implantation	<2.2e−16	0.93
Ovulation–LMP	<2.2e−16	0.91
LMP–CRL	<2.2e−16	0.88

**Fig. 5:**
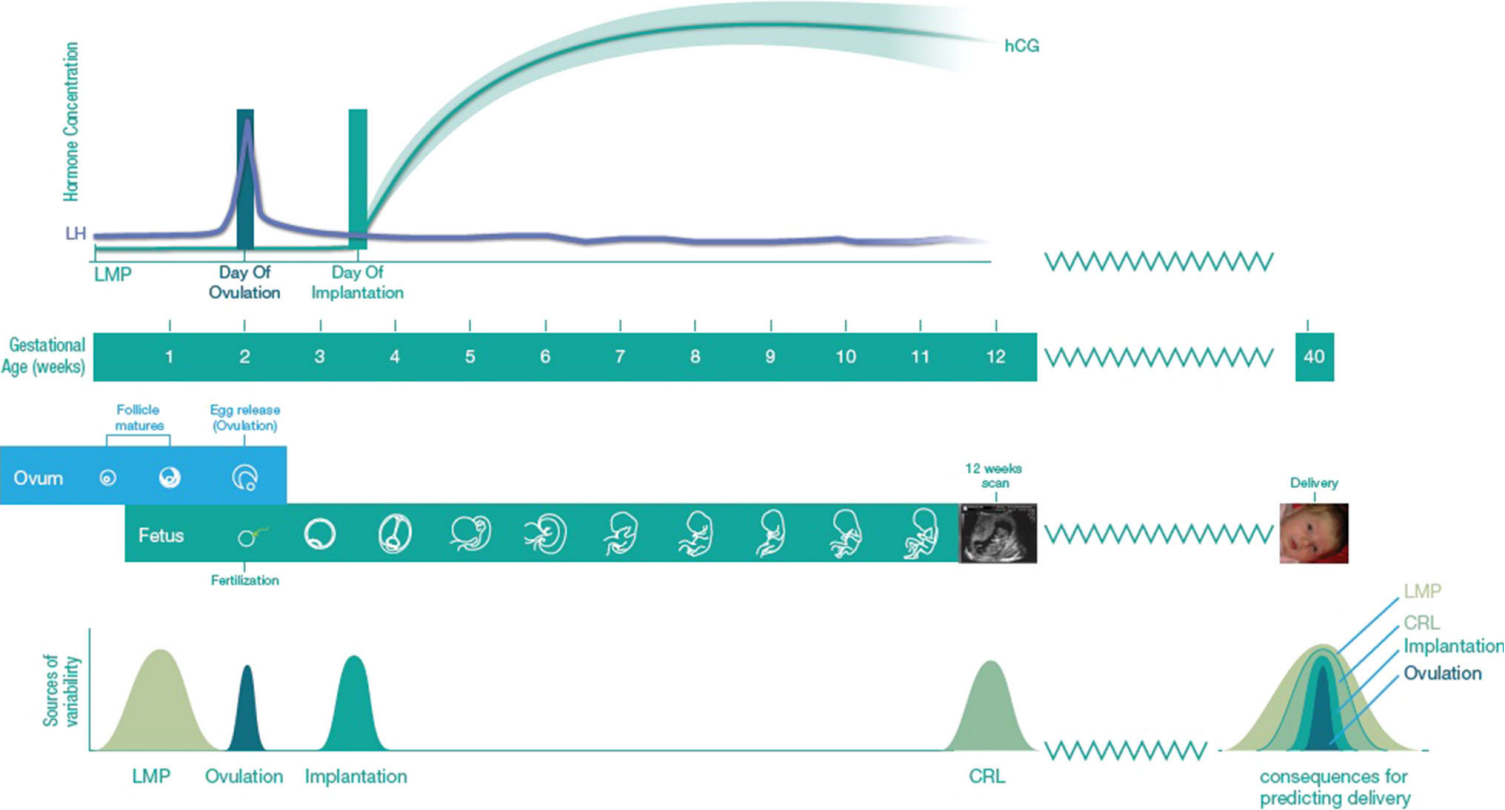
Figurative representation of the different methods of dating pregnancy and determination of gestational age

The relationships between the GA reference methods examined in our study and the implication of their associated variability for the prediction of delivery date are summarised in Fig. 5. With it being apparent that for the best estimate of delivery date and description of the natural history of human pregnancy duration, the most accurate reference method of GA being day of ovulation.

## Comment

We report that the gestational age distribution at delivery based on ovulation timing gives the narrowest frequency distribution for observed gestation at delivery and smallest mean difference. The converse is true for LMP and CRL, with implantation date somewhere in between. When the methods of GA assignment are compared in relation to observed length of gestation, implantation and ovulation day correlate most strongly, both LMP and CRL least strongly. When all methods are compared pair-wise, the widest limits of agreement where gestational length is determined from LMP vs ultrasound CRL, and narrowest where GA assessment is by ovulation vs implantation.

To our knowledge, this type of comparison of all four possible methods of assigning gestational age has not been performed before. The results challenge the conventional wisdom, though are supported by a study on women recruited in the 1980s, in which ovulation offered a less variable way of predicting gestational length than that derived from LMP [24]. Similarly, assignment of GA using ovulation reduced intra-individual variation in hCG rise in early pregnancy compared to assignment by CRL measurement or LMP [28]. However, neither study considered using implantation date to assign GA.

It has always been assumed that first trimester ultrasound measurement of CRL is the most accurate method of assigning GA, representing an important improvement over LMP, and hence ultrasound is the method that has been recommended for dating a pregnancy worldwide. Whilst this may be true for a population of woman amongst some of whom the LMP may not be known or there may be an inaccurate recollection, it may not hold where there is a known certain LMP. Ultrasound calculation of pregnancy duration has a measurement error, due to both inaccuracies in making the measurement, and the fact that not all fetuses grow at exactly the same rate [7, 29–31].

The magnitude of this error is widely reported, consistently being shown to be around 5 days. The study Verburg showed a median gestational age of 10 weeks that has 95 % confidence limits of 9 weeks 2 days to 10 weeks 6 days (in other words, a range of 11 days for 95 % of the data) [32]. Piantelli and colleagues also found the range to be 11 days at 12 weeks [33]. The commonly used Hadlock formula gives the 95 % confidence interval of a CRL measurement as ±8 % of the predicted age (i.e., ±5.5 days at 10 weeks gestation) [10]. The most commonly used formula preset on most ultrasound equipment—states that “CRL measurement can be used to estimate maturity to within ±4.7 days with 95 % confidence” [7]. The American College of Obstetrics and Gynecology guidelines for use of LMP and ultrasound to estimate gestational age acknowledge that variability is associated with an ultrasound measurement [34]. Therefore, to avoid confusion caused by changing the LMP date, because the scan date is different, guidelines have recommended that LMP date can be kept if it is within the ultrasound’s variability, which is ±5 days for a dating scan.

Quite separately from the sources of error in making the ultrasound measurement, the Robinson and Fleming CRL charts do not account for a woman’s ovulation day [7]. As this is reported to occur later than day 14 of the menstrual cycle [13, 14], any first trimester CRL measurement, by assessing fetal size, takes account of differences in ovulation and implantation timing [20]. However, it is possible that an incorrect assumption of ovulation timing is inherent in the original construction of the CRL charts currently available and that this same assumption also underlies GA assessment based on LMP. Systematic bias has been demonstrated in several CRL conversion formulas, for example, the Hadlock formula overestimates GA by 2–3 days [11, 28]. It is plausible that using CRL to assign gestation may be less accurate than certain LMP. Conversely, of course, using CRL would almost certainly be more accurate where LMP was unknown or uncertain.

The results of this study cannot, therefore, be extrapolated to form guidance for an unselected population: the women that we recruited were all keeping accurate day-by-day menstrual records, hence giving LMP GA assessment the best chance of performing as well as possible. Where the error of using LMP to assign gestation may be in recollection by the woman, CRL, ovulation, and implantation timing are all prone to other forms of error.

Ovulation day is least prone to variability: the LH assay used in this study has been compared to ultrasound-observed ovulation and the surge was found to be on the day of ovulation in 15 % of cycles, be 1 day prior in 76 % of cycles, 2 days prior in 6 % of cycles, and more than 2 days in 3 % of cycles. Ovulation testing, hence, has a variability of ±1 day [35]. Embryonic implantation is reflected by a detectable rise in HCG in maternal urine, however, compared to current laboratory serum tests which have sensitivities of 0.01 mIU/ml, and the sensitivity of home urine tests means that hCG is not detected for up to 3–4 days after its first possible detection in urine. In addition, establishment of contact between the embryo and endometrium and time taken for excretion from maternal blood to urine mean that detection of urinary hCG is always a post-implantation event.

Though this is a relatively small cohort of women, the results are robust particularly in the context of other studies of this nature, including the seminal works by Wilcox [19] and Jukic [24] reporting on 140 women. Furthermore, the ‘standard’ CRL chart was derived from observations on only 81 women [7]. We did not censor the cases where delivery was not spontaneous, as we sought to compare gestational length by different methods in the same group of women, and hence this is unlikely to have confounded the correlation or Bland–Altman analyses of agreement. This study also only considered normal, healthy women, and extrapolation to a wider population where pre-existing conditions may affect fetal growth, greater variability may be associated with CRL measurements; we would, in particular, caution against interpreting these findings in the context of assisted conception.

In summary, widely held assumptions on GA assignment may not be robust. The implication of these findings is that the most accurate methods of assigning GA, hence predicting length of gestation, are those based on ovulation or implantation, whereas the least accurate examined in this study are those based on LMP and ultrasound CRL. Where ovulation date is known in spontaneous conception, this may, in fact, be the most accurate method of dating. More fundamentally, as the prediction of gestational length by ovulation and implantation is most strongly correlated, gestational length is probably defined by a relatively fixed time after the embryo has implanted rather than time from LMP, especially where ovulation timing diverges from what is expected in a regular cycle. Knowledge of day of ovulation in a cycle, in which conception occurs, if available, appears from this study to provide the most reliable estimate of gestational age.
